# Human Papillomavirus Infection at the Time of Delivery

**DOI:** 10.7759/cureus.15364

**Published:** 2021-06-01

**Authors:** Mihaela C Radu, Calin Boeru, Melania-Elena Pop-Tudose, Andrei Necsulescu, Anca Dumitrescu, Claudia F Iancu, Irina Nita, Alexandra M Limbau, Corneliu Zaharia

**Affiliations:** 1 Birth Block, Obstetrics and Gynecology Hospital, Ploiesti, ROU; 2 Obstetrics and Gynaecology, Carol Davila University of Medicine and Pharmacy, Bucharest, ROU; 3 Emergency Room, Central Military Emergency Universitary Hospital "Dr. Carol Davila”, Bucharest, ROU; 4 Physics and Pharmauceutical Informatics Discipline, Carol Davila University of Medicine and Pharmacy, Bucharest, ROU; 5 Nursing, Carol Davila University of Medicine and Pharmacy, Bucharest, ROU; 6 Oncology, Elias Universitary Emergency Hospital, Bucharest, ROU; 7 Dermatology, Municipal Hospital, Curtea de Arges, ROU; 8 Biophysics Laboratory, Stefan S Nicolau Institute of Virology, Bucharest, ROU

**Keywords:** torch, hpv infection, newborn, human papilloma virus, pregnancy

## Abstract

Human papillomavirus (HPV) is one of the most encountered viral etiologies of genital infections that are transmitted through the sexual route in sexually active females. In the genital area, condylomata acuminate warts and the Buschke-Loewenstein tumor (giant condyloma acuminatum) are described. These lesions are associated with benign HPV6 and HPV 11 types. Condylomata acuminate may appear as exophytic growth similar to a cauliflower and is usually asymptomatic. The Buschke-Loewenstein tumor appears as ulcerated cauliflower-like lesions, often associated with fistulas and abscesses. They present exophytic and endophytic growth, local invasion, and high recurrence rates. This type of lesion may be associated with malignant HPV types. Here we present the case of a 34-year-old year pregnant woman who presented herself at the emergency room in labor with no previous medical evaluation during the pregnancy. The local examination revealed normal pubic hair, vulvar hyperpigmentation, and tonic and continent anal sphincter. At the vulvar level, a bulky cauliflower-like formation appeared. All routine investigations were normal. Immunological tests revealed the presence of antibodies anti-HPV immunoglobulin M (IgM) and immunoglobulin G (IgG). Treponema pallidum hemagglutination (TPHA) and HIV tests were negative. Samples collected from the genital lesions tested positive for both 6 and 11 DNA/HPV. The patient was diagnosed with condylomata acuminate and C-section was indicated as the methodology of birth so HPV infection of the newborn was avoided. We believe that HPV infection during pregnancy must be documented and treated when detected in order to avoid transmitting it to the newborn baby in a manner similar to TORCH testing. In pregnant women and women that want to conceive, in order to avoid transmission of infectious diseases from the mother to the newborn baby, TORCH testing is recommended. TORCH represents an acronym that includes: toxoplasmosis, other infectious diseases, rubella, cytomegalovirus infection, and herpes simplex infection.

## Introduction

Human papillomavirus (HPV) is one of the most encountered viral etiologies of genital infections that are transmitted through the sexual route in sexually active females [1-4]. More than 200 types of HPV have been identified and different classifications according to tropism and oncogenic risk have been issued. There are five genera: alpha, beta, gamma, mu, and nu. Each genus comprises several species and each species includes certain types.

According to the malignant potential of HPV in the genital area, there are three risk categories: low-risk HPV types (6, 11, 40, 42, 43, 44), intermediate-risk HPV types (31, 33, 35, 51, 52), and high-risk HPV types (16, 18, 45, 56) [5-6]. In the genital area, condylomata acuminate is often described. It may appear as exophytic growth similar to a cauliflower and is usually asymptomatic. The low-risk HPV 6 and HPV 11 are responsible for it [7]. Besides condylomata acuminate and warts, around the genital area, the Buschke-Loewenstein tumor (giant condyloma acuminatum), other benign lesions associated with HPV6 and HPV 11, is also encountered. This benign tumor appears like ulcerated cauliflower-like lesions, often associated with fistulas and abscesses. They present exophytic and endophytic growth, local invasion, and high recurrence rates [8]. This type of lesion may be associated with malignant HPV types.

Human papillomavirus is a virus that may be transmitted not only through close skin-to-skin contact and sexual contact but also through natural birth. We present a case of HPV genital infection in a pregnant woman at the moment of delivery.

## Case presentation

Our patient presented herself at the emergency room (ER) in labor. She had no previous medical evaluation during the pregnancy. The patient was a 34-year-old year woman, not married. Her personal physiological history revealed: menarche at 12 years, regular menstrual cycle at 28 days with an average duration of three to five days, a moderate menstrual flow of about three to four absorbents per day, and no phenomena associated with menstruation. The woman had no previous birth but she suffered two abortions, one on request and one spontaneous at eight gestational weeks. Her personal pathological history revealed one appendectomy when she was 13 years old. She presented no family diseases or chronic illnesses. The living and working conditions were poor; her average monthly income was low. The patient denied alcohol intake, she smoked about 10 cigarettes per day for 11 years, and she drank one cup of coffee per day. She had no stable partner.

**Figure 1:**
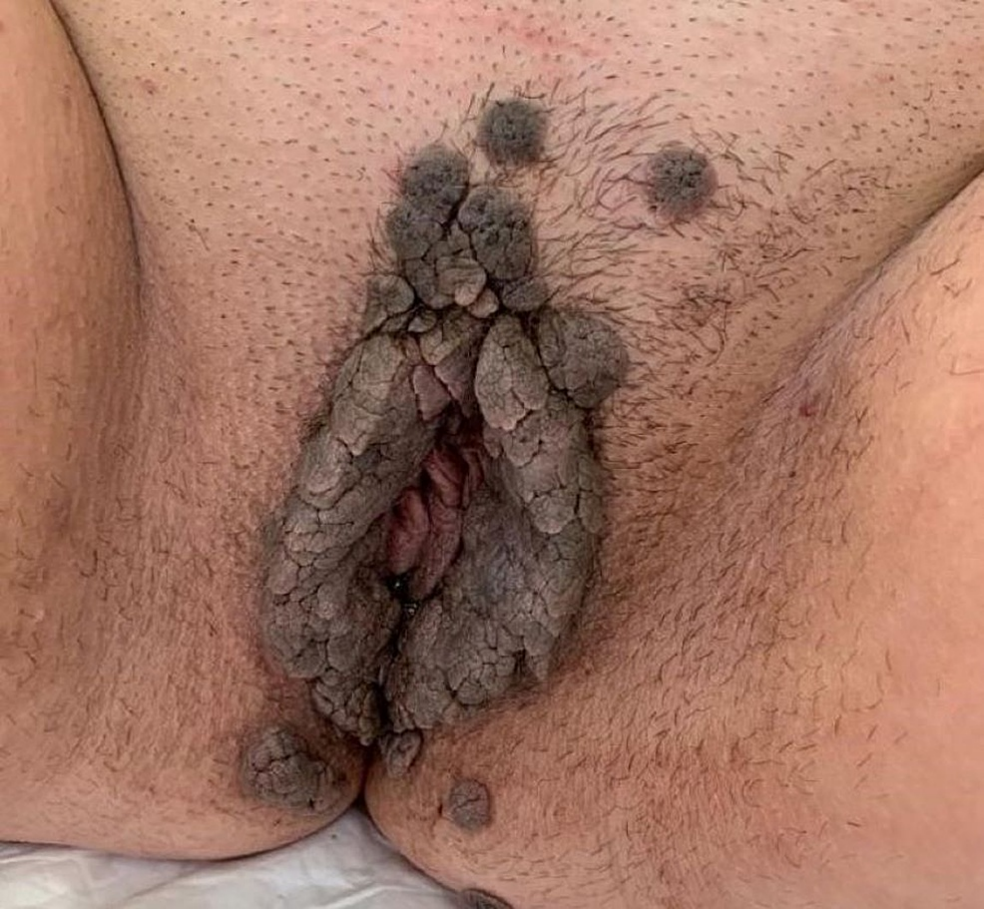
Aspect of the genital lesions at the moment of birth.

The local examination revealed normal pubic hair, vulvar hyperpigmentation, and tonic and continent anal sphincter. At the vulvar and vaginal level, a bulky cauliflower-like formation appeared (Figure 1).

Inside the vagina, abnormal and uncharacteristic leucorrhea appeared in a moderate amount. The cervix presented 2-3 cm dilation, the membranes were intact, and the fetus was in normal cephalic presentation. The patient presented 20 seconds long painful uterine contractions every five minutes. The fetal heartbeat was 146 beats per minute.

Ultrasound examination revealed: intrauterine pregnancy with single live fetus in cephalic (left occipito-iliac) presentation, a biparietal diameter of 89 mm, a cranial circumference of 317 mm, an abdominal circumference of 315 mm, and a femur length of 67 mm. The amniotic fluid was in normal amount, the amniotic fluid index was 15 cm, the estimated fetal weight was 2500 g, and the estimated gestational age was 37 weeks. The fetus was symmetrically developed, cardiac activity was present, fetal heartbeat was within normal limits, the placenta was inserted anterior and fundic. All information, anamnestic data, clinical and paraclinical examinations supported the diagnosis: 37 weeks pregnancy with live fetus in cephalic presentation, intact membranes, and voluminous vulvar condylomatosis.

Routine biochemical and hematological investigations were normal. Immunological tests revealed the presence of antibodies anti-HPV immunoglobulin M (IgM) and immunoglobulin G (IgG). Treponema pallidum haemagglutination (TPHA) and HIV tests were negative. After the presumption diagnosis of the genital lesion was ruled out, C-section was indicated as the methodology of birth for avoiding HPV transmission at the moment of birth.

After the birth, the etiology of the genital lesion was established by molecular diagnosis. DNA was extracted from 200 µL COPAN media mixed with the biological sample, using the QIAmp DNA Minikit (QIAgen, France SAS, Courtaboeuf Cedex) commercial test, according to the manufacturer’s instructions. The concentration and purity of each DNA were evaluated using NanoDrop spectrophotometer (NanoDropTechnologies, Montchanin, DE, USA) while the integrity was confirmed by a polymerase chain reaction (PCR) for a 110 bp β-globin gene fragment using PC03/ PC04 primers. For HPV detection and typing commercially available INNOLIPA (Innogenetics NV, Gent, Belgium) kit based on the reverse hybridization principle was used. This assay amplifies a broad spectrum of HPV types but allows detection of 16 different genotypes (16, 18, 31, 33, 45, 51, 58, 59, 68 ⁄73, 53, 66, 70, 6, 11, 40, 54). The kit is designed for the identification of different HPV genotypes by detection of specific sequences in the L1 region of the HPV genome. Part of the L1 region of the HPV genome is amplified, and the resulting biotinylated amplicons are then denatured and hybridized with specific oligonucleotide probes. These probes are immobilized as parallel lines on membrane strips. After hybridization and stringent washing, streptavidin-conjugated alkaline phosphatase is added, which binds to any biotinylated hybrid previously formed. Incubation with 5-bromo-4-chloro-3-indolyl phosphate/nitro blue tetrazolium (BCIP/NBT) chromogen yields a purple precipitate and the results can be interpreted according to the positive and negative controls. Samples are classified as positive for “any HPV” if at least one HPV type was detected by genotyping. In our sample, both six and 11 were detected so the clinical presumption was confirmed. Condylomata acuminate was the diagnosis for the genital lesion.

## Discussion

Human papillomavirus is an important etiology to be revealed in pregnancy for two reasons. One is the fact that genital infections may affect the process of conception or even the outcome of pregnancy. The other is that the infection with HPV of the placenta is possible because trophoblastic cells appear to have mechanisms for HPV replication [9-10]. The placental damage can directly cause fetal growth retardation, preeclampsia, abortions, and preterm birth.

High-risk HPV types may be involved in cellular transformation and different types of cancers [11-12].

The Center for Disease Control and Prevention (CDC) recommends that pregnant women get two vaccines during every pregnancy: the inactivated flu vaccine (the injection, not the live nasal flu vaccine) and the Tdap vaccine [13-14]. Pregnant women are also encouraged to get vaccinated anti-COVID-19 [15]. On the other side, HPV vaccination is not recommended during pregnancy.

In pregnancy, due to the changes that occur, the maternal body is in a stage of physiological immunosuppression, being more prone to contract infections. In pregnancy prenatal HPV transmission is vertical transmission from mother to fetus and at birth, perinatal HPV transmission is vertical transmission from mother to newborn, a hypothesis supported by the fact that the viral types identified in the mother were the same as those identified in the newborn [16]. Transmission can also occur at the time of fertilization, at the time of conception, through the infected sperm, the virus being identified in semen and seminal fluid in a variable proportion of 8%-64% [17]. Transmission is the most common during spontaneous birth, being described as the classic mechanism of contracting the infection [18-19].

Our patient presented low-risk HPV types six and 11 and was diagnosed with condylomata acuminate, initially by the clinical appearance and later by the identification of the HPV types.

The C-section was performed and a possible HPV transmission to the newborn was avoided. After the birth, the woman was treated and the HPV lesion was surgically removed. The newborn was under the family doctor's supervision until the age of one year. No signs of HPV transmission were detected.

## Conclusions

Clinical HPV infection in the antepartum period may be documented and treated in the same manner as the other infections that can be transmitted from the mother to the newborn or fetus, toxoplasmosis, other infectious diseases, rubella, cytomegalovirus infection, and herpes simplex infection (TORCH) infections. TORCH infections classically comprise toxoplasmosis, Treponema pallidum, rubella, cytomegalovirus, herpes virus, hepatitis viruses, HIV, and other infections. These infections are major contributors to prenatal, perinatal, and postnatal morbidity and mortality. The effects of HPV on pregnancy and the transmission to the newborn baby are not completely understood and further studies are needed.
